# The Treatment of Whooping Cough

**Published:** 1908-07-25

**Authors:** Edmund Hughes


### THE TREATMENT OF WHOOPING COUGH.

By EDMUND HUGHES, M.R.C.S.Eng., L.R.C.P.Lond.

A NUMBER of new remedies for whooping-cough have been suggested of late years, and the main object of this article is to bring these together in a convenient way. After the end of the first week the child may be provided with an abdominal binder, which is to be worn night and day throughout, and is in very many cases valuable in limiting the paroxysms and preventing vomiting. If the binder is to succeed it must be well fitting and sufficiently tight. Hospital out-patients may have a muslin or partly woollen material adjusted on the spot by a nurse, but the better plan is to have a belt made of flannel or linen front and back with some slightly yielding material, as elastic webbing or stockinette, let into the sides, the back being laced up to the degree of tightness necessary for control of symptoms without undue constriction. Dr. T. W. Kilmer (New York) advises the width to be from four to eight inches, according to age, the length three inches less than the abdominal girth at the navel. These details are important, for the success of the binder probably depends on their being followed.

For drugs an innovation is tincture of *Grindelia robusta*, given in half-drachm doses in sweetened water four times daily. Two fluorine compounds have had favourable notice—*i.e.* antitussin ointment (one daily inunction of about ʒss), and solution of fluoroform, a drug whose use in pertussis was reported on this year by Tessier in France, but which needs further testing. If a nightly sedative is found necessary, codeine (gr. $\frac{1}{12}$-$\frac{1}{8}$), heroin (gr. $\frac{1}{25}$-$\frac{1}{15}$), or chloral 5 to 10 grains in enema may be tried. Other recent combinations are codeine or morphine gr. $\frac{1}{40}$ in syrup of tolu ʒj., from ʒss.-ʒj. being given three or four times daily; and this dose may be combined with 1, 2, or even 3 grains of antipyrin after the second year, according to age and severity.

The above measures, together with a diet limited in starch and excluding foods likely to irritate the pharynx (such as gritty preparations and acids in food or drink), seclusion from non-infected children, and abundance of fresh air, summarise modern ideas in uncomplicated cases. But there are three or four further suggestions in recent literature that call for notice. One is vaccination. The possible efficacy of this has been known for some years, but latterly a fresh series of cases has forced it on the practitioner's attention as worth a trial in obstinate cases. Another is chloroform narcosis for twenty minutes to full surgical degree. Only a few days' effect is claimed for this and in some cases, but the results are so far contradictory. In one case of the writer's symptoms recurred with their former vehemence as soon as the patient had recovered from the immediate effect of the anæsthetic, but four days later the cough, which hitherto had been extremely severe and prostrating, rapidly abated, and afterwards hardly needed treatment. The latest suggestion of all is to give the milk of animals which have been injected with antitetanic serum (for particulars see *Lancet*, June 20, 1908).

Though formalin vapour and carbolic acid inhalations are of doubtful value, iodide of ethyl vapour may be inhaled with the object of mitigating cough. Oxygen inhalation is especially called for when cyanosis is so marked and persistent that convulsions or lung-collapse have to be feared. It is most effectively administered at night.

When the spasmodic stage is nearly ended a prescription commended by Dr. Eustace Smith may be tried—tinct. cantharidis ♏ij. with 5 minims each of paregoric and tincture of cinchona ter die. Much may be done to hasten convalescence by a close attention to the digestive tract. A frequent late syndrome is complete loss of appetite with a slimy tongue, great restlessness at night, and intermittent looseness of the bowels. This may often be most effectively met by a mixture of sod. bicarb. gr. vj.-xij. in infusum rhei ʒij.-iij. three times daily between meals, followed in a few days by a mixture of tincture of hydrastis, sod. bicarb., gentian and an aromatic, and a small dose of calomel every second or third night. With these a diet should be arranged so as to include a minimum of sugar and starch for weeks together.

